# A Tumor of Intrigue: Undifferentiated Carcinoma With Osteoclastic Giant Cells of the Urinary Bladder

**DOI:** 10.7759/cureus.114249

**Published:** 2026-08-10

**Authors:** Sampa Choudhury, Sithara Aravind, Sangeetha K Nayanar, Vivek Viswanath

**Affiliations:** 1 Department of Oncopathology, Malabar Cancer Centre, Thalassery, IND; 2 Department of Surgical Oncology, Malabar Cancer Centre, Thalassery, IND

**Keywords:** bladder, giant-cell tumor, hematuria, osteoclasts, rare diseases

## Abstract

Undifferentiated carcinoma with osteoclastic giant cells (UCOGC) of the urinary bladder is a recently described entity classified as a subtype of poorly differentiated urothelial carcinoma, which is a form of invasive urothelial carcinoma. It is an extremely rare and aggressive malignancy that occurs predominantly in elderly men. The rarity of this neoplasm and its morphological similarity to other giant cell-rich lesions may present diagnostic challenges. Here, we present a case of urinary bladder carcinoma with a biphasic appearance. Histomorphological examination and immunohistochemical studies are important for identifying this entity and differentiating it from other carcinomas with similar features, given the significant prognostic and therapeutic differences among these entities.

## Introduction

Undifferentiated carcinoma with osteoclastic giant cells (UCOGC) of the urinary bladder is an extremely rare and aggressive entity of unknown pathogenesis. Several terms have been used in the literature to describe this condition, including osteoclast-rich undifferentiated carcinoma, osteoclast-like giant cell carcinoma, and giant cell tumor (GCT)-like lesion of the bladder [1-3]. In the 5th edition of the World Health Organization (WHO) Classification of Tumours of the Urinary Tract and Male Genital Tract (2022), this entity is included under the poorly differentiated urothelial carcinoma subtype. It is predominantly seen in elderly patients, with a male predominance [4]. The clinical symptoms are nonspecific and usually include gross hematuria and flank pain. Histologically, it is a biphasic tumor that resembles a GCT, although these two entities differ in both biological behavior and management. Similar histological tumor types have also been reported in other organs, including the liver, gallbladder, pancreas, breast, intestine, salivary glands, thyroid gland, female genital tract, and skin, as well as in the renal pelvis, ureter, and kidney of the urinary system [1,3].

Here, we present the case of an 86-year-old man with UCOGC of the urinary bladder. To the best of our knowledge, this is the first reported case of this type from southern India. Furthermore, to assist pathologists in establishing an accurate diagnosis, we have outlined a diagnostic algorithm for urothelial carcinomas with multinucleated giant cells.

## Case presentation

**Figure 1:**
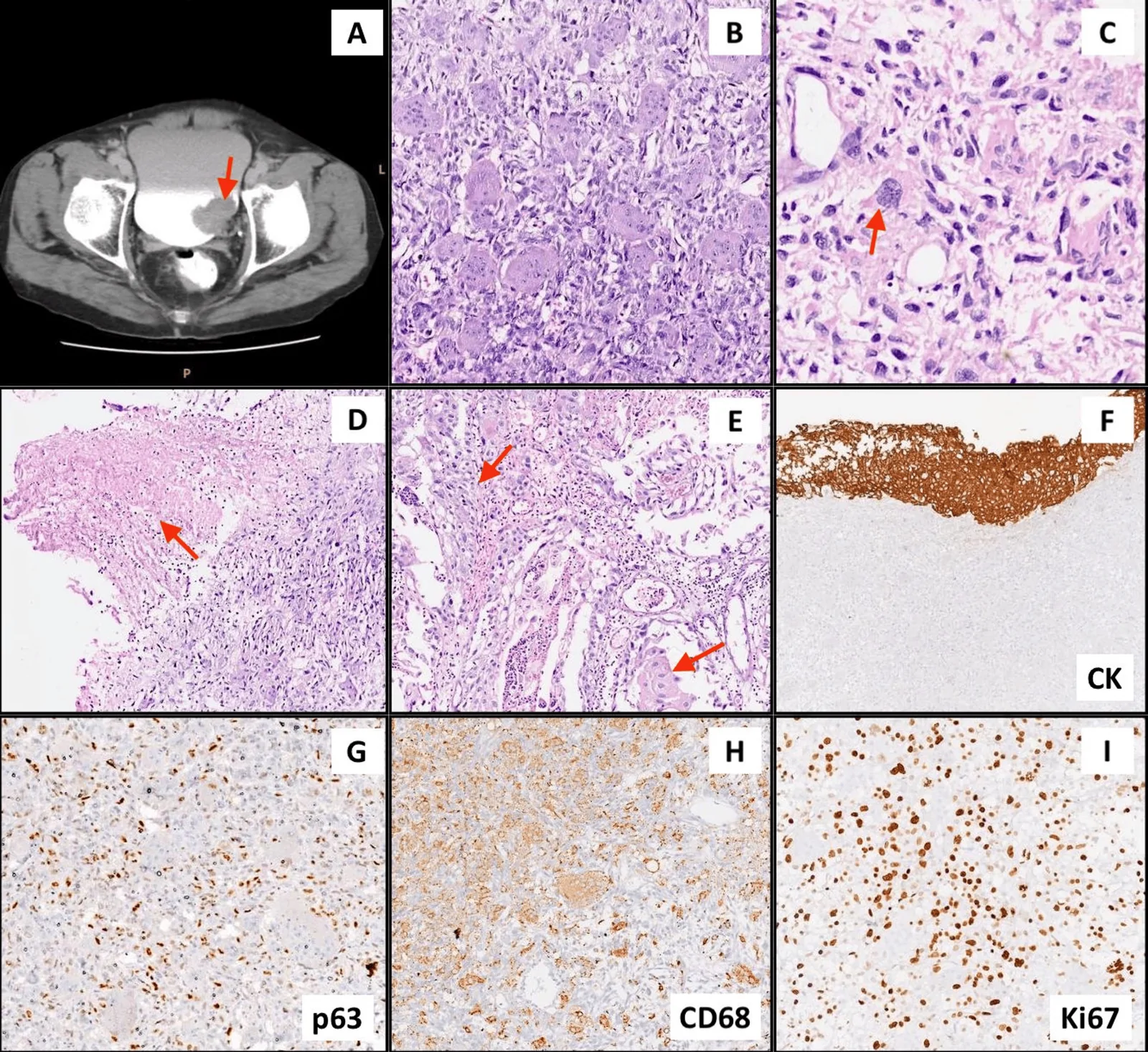
Radiological and histopathological findings (A) CECT scan shows polypoid growth in the urinary bladder (red arrow). (B) Mononuclear tumor cells and osteoclastic giant cells. (C) Focal marked pleomorphism (red arrow). (D) Focal necrosis (red arrow). (E) Conventional urothelial carcinoma (red arrow) (hematoxylin & eosin, 400x). (F-I) Immunohistochemistry (IHC) for cytokeratin (CK) stains the urothelium, whereas both mononuclear tumor cells and giant cells are negative. Mononuclear cells are positive for p63. Giant cells are positive for CD68, and Ki-67 index is high (IHC, 400x).

An 86-year-old man with a history of diabetes mellitus and bronchial asthma presented with episodes of gross hematuria. He had been a chronic smoker for the past 30 years. On examination, he had multiple bilateral cervical and left supraclavicular lymphadenopathies. Contrast-enhanced computed tomography (CECT) revealed a 4 × 3 × 3 cm polypoid growth arising from the posterolateral wall of the urinary bladder and involving the left vesicoureteric junction (Figure 1A). Enlarged external iliac and para-aortic lymph nodes were also noted. His Eastern Cooperative Oncology Group (ECOG) performance status was 2.

The patient underwent transurethral resection of bladder tumor (TURBT) for histopathological examination. Microscopically, fragmented bits of neoplastic urothelial cells were arranged in papillary architecture, with focal invasion into the muscularis mucosae. A few fragments showed singly scattered and haphazardly arranged mononuclear cells in the lamina propria, exhibiting marked pleomorphism, hyperchromatic nuclei, irregular nuclear membranes, and scant cytoplasm. Numerous osteoclastic giant cells were dispersed among the mononuclear cells. Focal areas showed bizarre, pleomorphic mononuclear cells and tumor necrosis. The mitotic count was 15-20 per 10 high-power fields (HPFs) (Figure 1B-1E). No significant inflammation, granuloma, or sarcomatous component was identified. The detrusor muscle was free of tumor, consistent with non-muscle-invasive bladder cancer (NMIBC). A provisional diagnosis of a poorly differentiated malignant neoplasm was made, and a panel of immunohistochemical (IHC) markers, including epithelial markers (cytokeratin, p63, and GATA3) and a histiocytic marker (CD68), was performed. Cytokeratin (CK) was positive in the neoplastic urothelial cells but negative in the mononuclear and giant cells. p63 was positive in both the urothelial and mononuclear cells but negative in the giant cells. The giant cells showed strong positivity for CD68. The proliferative index (Ki-67) was 60%-70% in hotspot areas (Figure 1F-1I). GATA3 was inconclusive despite repeated attempts.

In view of the characteristic histomorphological features and immunoprofile, a final diagnosis of UCOGC was made. The estimated clinical TNM stage at the time of presentation was cT1N2M1a, according to the Union for International Cancer Control (UICC) 9th edition. Cervical lymph node biopsy and further imaging were planned for appropriate tumor staging; however, the patient declined further medical evaluation, was subsequently lost to follow-up, and ultimately died six months later.

## Discussion

From a histological perspective, the UCOGC bears resemblance to GCT of the bone or tendon sheath, consisting of mononuclear neoplastic cells interspersed with many osteoclast-like multinucleated giant cells. Nevertheless, these two entities exhibit different biological behavior, as GCT is a benign locally aggressive neoplasm with a favorable prognosis, whereas UCOGC is an aggressive neoplasm with a dismal prognosis [5]. It is frequently associated with high-grade/low-grade urothelial carcinoma and/or carcinoma in situ of the surface epithelium. The mononuclear cells are oval to spindle shaped with vesicular nuclei, mild to moderate pleomorphism, and frequent mitoses, including atypical mitoses. On the other hand, the giant cells have banal morphology with no mitotic activity. Increased vascularization and focal areas of hemorrhage are also discernible [1,3].

The immunoprofile of mononuclear cells shows patchy to diffuse positivity for one or more epithelial markers, including pancytokeratin, EMA, GATA3, thrombomodulin, and p63. The multinucleated giant cells are positive for CD68, CD45, CD51, CD54, and vimentin and are negative for epithelial markers [5,6].

**Figure 2:**
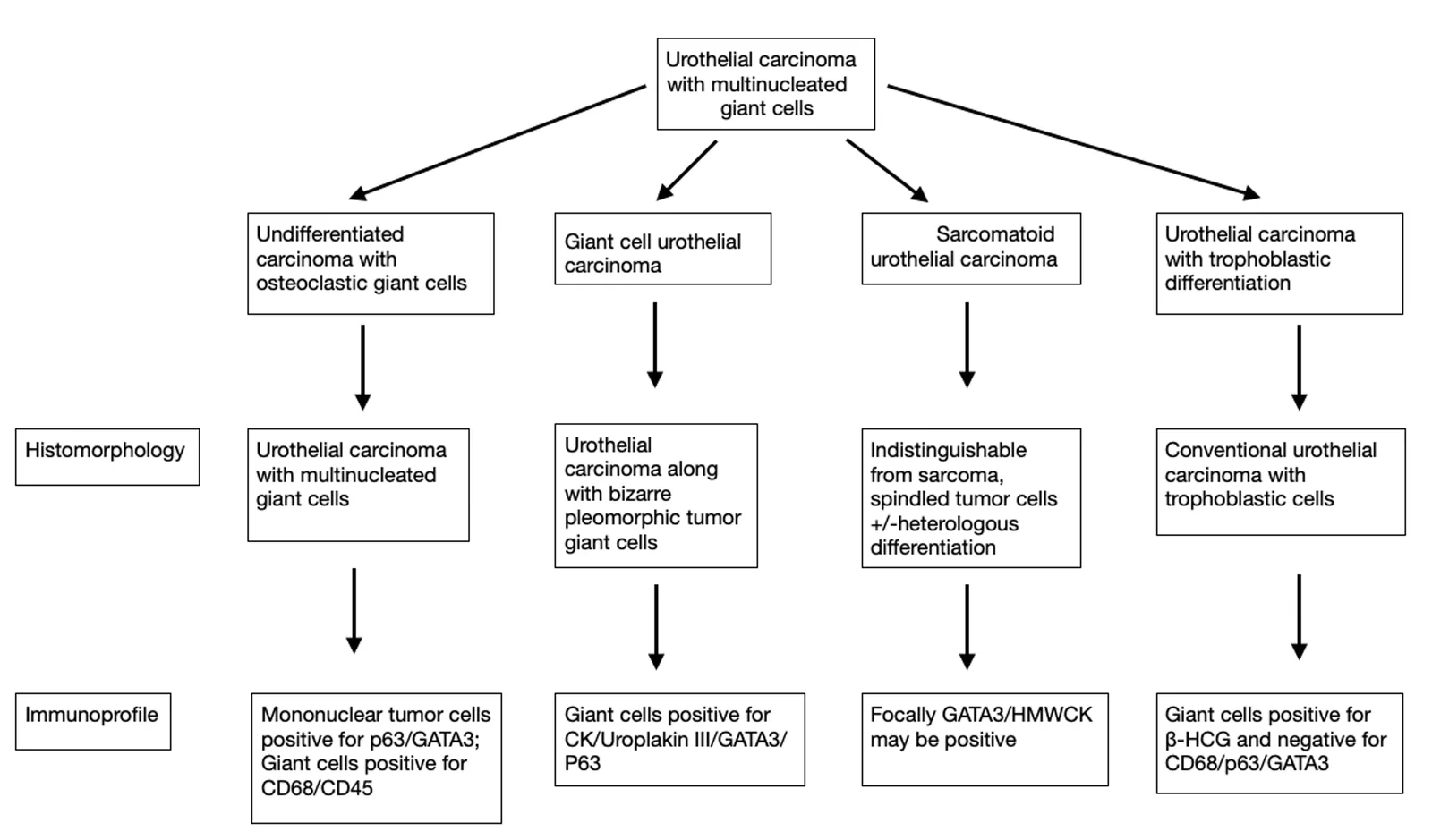
Flowchart depicting the differential diagnoses of urothelial carcinoma with multinucleated giant cells

The differential diagnoses include giant cell urothelial carcinoma, sarcomatoid urothelial carcinoma, and urothelial carcinoma with trophoblastic differentiation [1,4,7,8]. The differentiating points, including histomorphology and immunoprofile, are depicted in Figure 2.

The rarity of this disease has resulted in limited molecular analysis data. To date, three articles have reported genetic alterations associated with this entity in the urinary system, including mutations and copy number variations involving several genes, such as TERT promoter, PIK3CA, HRAS, KRAS, TP53, MDM2, CREBBP, KDM6A, ARID1A, ARAF, CDKN2A, and CDKN2C [9-11]. In our case, molecular information was limited because the patient declined further medical evaluation and investigations.

Similar histomorphological features and histiocytic characteristics of the osteoclast-like giant cells have been described in this entity occurring at other sites, including the pancreas, breast, and thyroid. The prognosis is variable, ranging from indolent to aggressive disease. Genetic alterations have been well described in pancreatic UCOGC, with reported mutations in KRAS, BRCA2, CDKN2A, TP53, SMAD4, and GNAS [10,12-14].

The National Comprehensive Cancer Network (NCCN) published revised guidelines for the management of NMIBC according to the American Urological Association (AUA)/Society of Urologic Oncology (SUO) risk stratification in 2021. UCOGC falls within the high-risk NMIBC category because of its aggressive histological characteristics. Radical cystectomy is the recommended treatment for bacillus Calmette-Guérin (BCG)-unresponsive cases or patients with very high-risk features. Intravesical chemotherapy or pembrolizumab are other preferred treatment options [15]. The prognosis is generally poor despite surgery and adjuvant chemotherapy. In advanced bladder cancer, cisplatin-based chemotherapy is the standard treatment. However, mutations in ARID1A, TP53, and MDM2 may contribute to chemotherapy resistance. The role of radiotherapy remains controversial. Overall survival is usually less than two years according to the available literature [2,10,16].

A tabular comparison of the present case with other published cases of UCOGC involving the urinary tract from 2000 to 2026, for which open-access full-text articles were available in the English-language literature, is presented in Table 1. A total of 55 cases were included in the comparison. Most patients were elderly men, and the urinary bladder was the most commonly involved site, with hematuria being the most common presenting symptom. The associated malignancies were predominantly urothelial carcinoma and/or carcinoma in situ (CIS). These findings from the literature are consistent with those observed in our present case. However, further investigations for tumor staging and subsequent management could not be performed because the patient declined further medical evaluation. The overall survival (OS) in the present case was comparable with that reported in other studies.

**Table 1:** Comparative analysis of the present case and documented cases of UCOGC involving the urinary tract reported in the English-language literature from 2000 to 2026 CIS: carcinoma in situ, EFS: event-free survival, F: female, M: male, NA: not available, OS: overall survival, TURBT: transurethral resection of bladder tumor, UC: urothelial carcinoma, UCOGC: undifferentiated carcinoma with osteoclastic giant cells.

Study	Number of cases	Age (year)	Gender	Previous history	Clinical features	Site	Size	Associated malignancies	Muscle invasion	Management	OS/EFS
Baydar et al. (2006) [1]	6	Range: 39-82 years. Mean age: 69.1 years	M	NA	Hematuria, flank pain, multiple pulmonary emboli	Renal pelvis (3); bladder (3)	Largest dimension: 11 × 7 × 2 cm	UC (4), CIS (4)	One case of bladder cancer	Radical nephroureterectomy for renal pelvis cancer; TURBT/radical cystectomy for bladder cancer	4-15 months OS
Park (2010) [2]	1	76	M	Chronic smoker	Hematuria, flank pain	Distal ureter	5 × 1.5 × 1.2 cm	UC	NA	Radical Nephroureterectomy	5 months EFS
Behzatoğlu et al. (2009) [3]	2	56, 74	M	NA	Hematuria	Bladder	NA	UC (1), none (1)	No	TURBT	35-42 months of EFS
Karasavvidou et al. (2022) [5]	1	62	M	Chronic smoker	Hematuria, hydronephrosis	Bladder	9.1 × 5.9 cm	None	Yes	TURBT, radical cystoprostatectomy, adjuvant chemotherapy	13 months OS, 7 months EFS
Priore et al. (2018) [6]	21	Range: 55-85 years. Mean age: 73 years	M (17); F (4)	NA	NA	Renal pelvis (6), bladder (15)	NA	UC (10), CIS (5), none (6)	NA	NA	NA
Satturwar et al. (2022) [9]	5	Range: 63-86 years. Mean age: 76 years	M	NA	Hematuria	Bladder	Largest dimension: 6 cm	UC (2), CIS (4)	Yes (2)	TURBT (3), radical cystoprostatectomy (2)	24 months OS (1), 36 months OS (1)
Kameyama et al. (2024) [10]	1	75	M	NA	Hematuria, pain on voiding urine	Bladder	Largest dimension: 5.6 cm	UC	Yes	TURBT, neoadjuvant chemotherapy, radical cystoprostatectomy	10 months EFS
Rizkalla et al. (2025) [11]	14	Range: 55-89 years. Mean age: 73 years	M (11), F (3)	NA	NA	Bladder (8), kidney (6)	NA	UC with other subtypes or differentiations (10)	NA	TURBT (7), nephrectomy/nephroureterectomy (6)	NA
Osman et al. (2019) [16] (Turkey)	1	55	M	Chronic smoker	Hematuria	Bladder	7 cm	Prostatic adenocarcinoma	Yes	Radical cystoprostatectomy, adjuvant chemotherapy	10 months OS
Wu et al. (2009) [17] (Taiwan)	1	62	M	NA	Hematuria	Bladder	NA	None	Yes	TURBT, Radical Cystoprostatectomy, partial ureterectomy	5 months EFS
Rana et al. (2021) [18]	1	63	M	Hypertension, hypertrophic cardiomyopathy, asthma	Hematuria	Bladder	3.2 × 2.6 cm	UC	Yes	TURBT	NA
Purohit et al. (2010) [19]	1	74	M	NA	Hematuria	Bladder	NA	UC, prostatic adenocarcinoma	Yes	TURBT, radical cystoprostatectomy	18 months EFS
Present case	1	86	M	Chronic smoker, diabetes, and asthma	Hematuria	Bladder	4 × 3 × 3 cm	UC	No	TURBT	6 months OS

## Conclusions

UCOGC is a rare and aggressive type of urothelial carcinoma with characteristic histomorphological features and immunoprofile. Although GCT of the bone shares microscopic features similar to those of UCOGC, the two entities have completely different biological behaviors; therefore, UCOGC should not be labelled as a GCT of the urinary bladder. Accurate identification of this entity and exclusion of its close mimickers are essential to facilitate appropriate clinical management.
